# Weighted Bayesian Belief Network for diabetics: a predictive model

**DOI:** 10.3389/frai.2024.1357121

**Published:** 2024-04-11

**Authors:** Shweta Kharya, Sunita Soni, Abhilash Pati, Amrutanshu Panigrahi, Jayant Giri, Hong Qin, Saurav Mallik, Debasish Swapnesh Kumar Nayak, Tripti Swarnkar

**Affiliations:** ^1^Department of Computer Science and Engineering, Bhilai Institute of Technology, Durg, Chhattisgarh, India; ^2^Department of Computer Science and Engineering, Siksha ‘O' Anusandhan (Deemed to be University), Bhubaneswar, Odisha, India; ^3^Department of Mechanical Engineering, Yeshwantrao Chavan College of Engineering, Nagpur, India; ^4^Department of Computer Science and Engineering, University of Tennessee at Chattanooga, Chattanooga, TN, United States; ^5^Department of Environmental Health, Harvard T H Chan School of Public Health, Boston, MA, United States

**Keywords:** diabetes disease prediction, Bayesian Belief Network, association rule mining, Weighted Bayesian Confidence, Weighted Bayesian Lift

## Abstract

Diabetes is an enduring metabolic condition identified by heightened blood sugar levels stemming from insufficient production of insulin or ineffective utilization of insulin within the body. India is commonly labeled as the “diabetes capital of the world” owing to the widespread prevalence of this condition. To the best of the authors' last knowledge updated on September 2021, approximately 77 million adults in India were reported to be affected by diabetes, reported by the International Diabetes Federation. Owing to the concealed early symptoms, numerous diabetic patients go undiagnosed, leading to delayed treatment. While Computational Intelligence approaches have been utilized to improve the prediction rate, a significant portion of these methods lacks interpretability, primarily due to their inherent black box nature. Rule extraction is frequently utilized to elucidate the opaque nature inherent in machine learning algorithms. Moreover, to resolve the black box nature, a method for extracting strong rules based on Weighted Bayesian Association Rule Mining is used so that the extracted rules to diagnose any disease such as diabetes can be very transparent and easily analyzed by the clinical experts, enhancing the interpretability. The WBBN model is constructed utilizing the UCI machine learning repository, demonstrating a performance accuracy of 95.8%.

## 1 Introduction

The global prevalence of diabetes is on the rise, emerging as a significant and pressing public health concern in the 21st century. Diabetes, characterized by either deficiency of insulin secretion, is a widespread chronic condition. According to recent statistics presented by the International Diabetes Federation (IDF), 8.8% of individuals affected by diabetes are approximately in the age range of 20–79 years. Notably, 46.5% of these cases go undetected, contributing to the alarming fact that ~5 million deaths annually are attributed to diabetes. Projections indicate that by 2040, the global diabetic population will reach 642 million (Ershadi and Seifi, 2020).

In the past few years, the exploration of medical data collected through data mining and machine learning techniques has captured the attention of numerous researchers. Researchers have worked with the Bayesian Belief Network due to its nature and suitability in the clinical world (Kharya et al., 2022). In the clinical sector, the Bayesian network projects conditional dependencies and independencies between the various symptoms of illness (Fazel Zarandi et al., 2018; Braik et al., 2023a). For example, “physical inactivity” may cause “obesity,” which may raise “blood glucose.” Moreover, BBN plays a significant role in understanding the association between symptoms. In this study, to improve the accuracy, BBN is reconstructed to incorporate one characteristic of the clinical world, i.e., “not all symptoms are equally important for prediction.” Every symptom has different predicting capabilities for any illness in the clinical world, for example, considering the symptoms of heart diseases such as chest pain, fainting, fatigue, shortness of breath, and swollen feet. Here, each symptom has a different impact on the severity of the disease. Therefore, it cannot be given equal weightage. According to the above example, chest pain impact is the highest among all the symptoms. Moreover, an innovative idea of assigning different weights to different symptoms based on the predictive capability is incorporated into this study. This study proposes a new concept, formula, and pseudocode to create a new Weighted Bayesian Belief Network (WBBN) model using a clinical dataset. In this model, the weighted concept is introduced and implemented with BBN.

The suitability of the BBN in the clinical domain as the best predictive computational model is presented in the study mentioned in the reference (Liu et al., 2018; Shen et al., 2018; Simsek et al., 2020; Braik et al., 2023b). After studying the impact of different disease symptoms, the major work is to find the correlation or association between the symptoms. The importance of the ARM method in discovering a correlation or association between attributes/symptoms is to predict the class label in the form of Class Association Rules (CARs). In conventional rule mining, the significance of a rule is typically determined by the count of item sets within a database. Traditional mining rules rely on support and confidence measures to identify frequent item sets, assuming that all items have equal significance (Topuz et al., 2018).

In contrast, social science, medical, and business market researchers hold distinct perspectives. A rule's significance is contingent on quantitative aspects, such as the frequency of an item in a database, and qualitative elements, involving human interpretation rather than solely relying on database metrics. Weights can be employed to depict the impact of symptoms within a dataset (Kumar et al., 2019).

This study introduces a novel classifier, WBBN, which employs Weighted Bayesian_class Association Rules (WBAR) to construct the computational model utilizing clinical datasets. This methodology initially allocates weights to various symptoms or attributes based on their predictive capacities. Next, the main focus is given to the WARM technique, which discovers the relationship between Weighted Two attributes and Weighted Multi attributes to represent hidden patterns and new knowledge. Then, the Weighted_class Association Rules (WAR) are extracted. The consequence of a rule is the 'class label' using interesting measures such as Weighted Support and Weighted Confidence on setting minimum threshold values. Bayesian theory is applied to the Weighted_class Association Rules to produce WBAR, utilizing Weighted Bayesian Confidence (WBC) and Weighted Bayesian Lift (WBL), which are employed to construct the WBBN. Subsequently, experiments were conducted on established clinical datasets to assess the accuracy of the performance of WBBN.

A brief literature survey is conducted on the research performed in the clinical sector of the last 5 years on different classifiers using the PIDD UCI machine learning dataset, as shown in Table 1. The classifiers considered are Naïve Bayes, Neural Network, Support Vector Machine, and Decision Tree. Accuracies of all the work are shown in Table 1. The literature survey shows that the Bayesian Belief Network classifier is a promising area to work on, and it shows better results when applied to the PIDD UCI machine learning dataset.

**Table 1:** A brief literature survey.

**References**	**Classifier**	**Year**	**Accuracy**
Resti et al. (2021)	Naïve Bayes	2021	93%
Chowdary and Kumar (2021)	Naïve Bayes	2021	87.3%
Jader and Aminifar (2022)	Artificial Neural Network	2022	91%
Bukhari et al. (2021)	Neural Network	2021	93%
Xie et al. (2017)	Bayesian Belief Network	2017	82.48%
Joseph et al. (2022)	Bayesian Belief Network	2022	92.2%
Patil et al. (2022)	Support Vector Machine	2022	94.5%
Hao et al. (2022)	Support Vector Machine	2022	95.92%
Azad et al. (2022)	Decision Tree & Genetic Algorithm	2022	82.12%
Abedini et al. (2020)	Ensemble Method	2020	83.08%

## 2 Datasets

Experiments are performed on clinical datasets such as diabetes using the Pima Indian Diabetics Dataset (PIDD) from the UCI machine learning data repository (Pima Indian Diabetes Dataset UCI.-ML Repository, 2023). The distribution and details of data are shown in Table 2.

**Table 2:** The UCI clinical machine learning dataset.

**Dataset**	**Size**	**Attributes**	**Class labels**	**Percentage of records in positive class label**	**Percentage of records in negative class label**
PIDD	768	9	2	35	65

The clinical datasets are acquired from the standard UCI archive. The discretized version is obtained through Liverpool University Computer Science-Knowledge Discovery in Data (LUCS-KDD). Discretization/Normalized (DN) software is utilized to convert data files from the UCI archive, which is an appropriate format for ARM applications. In this context, discretization refers to converting numeric attributes into categorical ones.

## 3 Methodology

**Figure 1:**
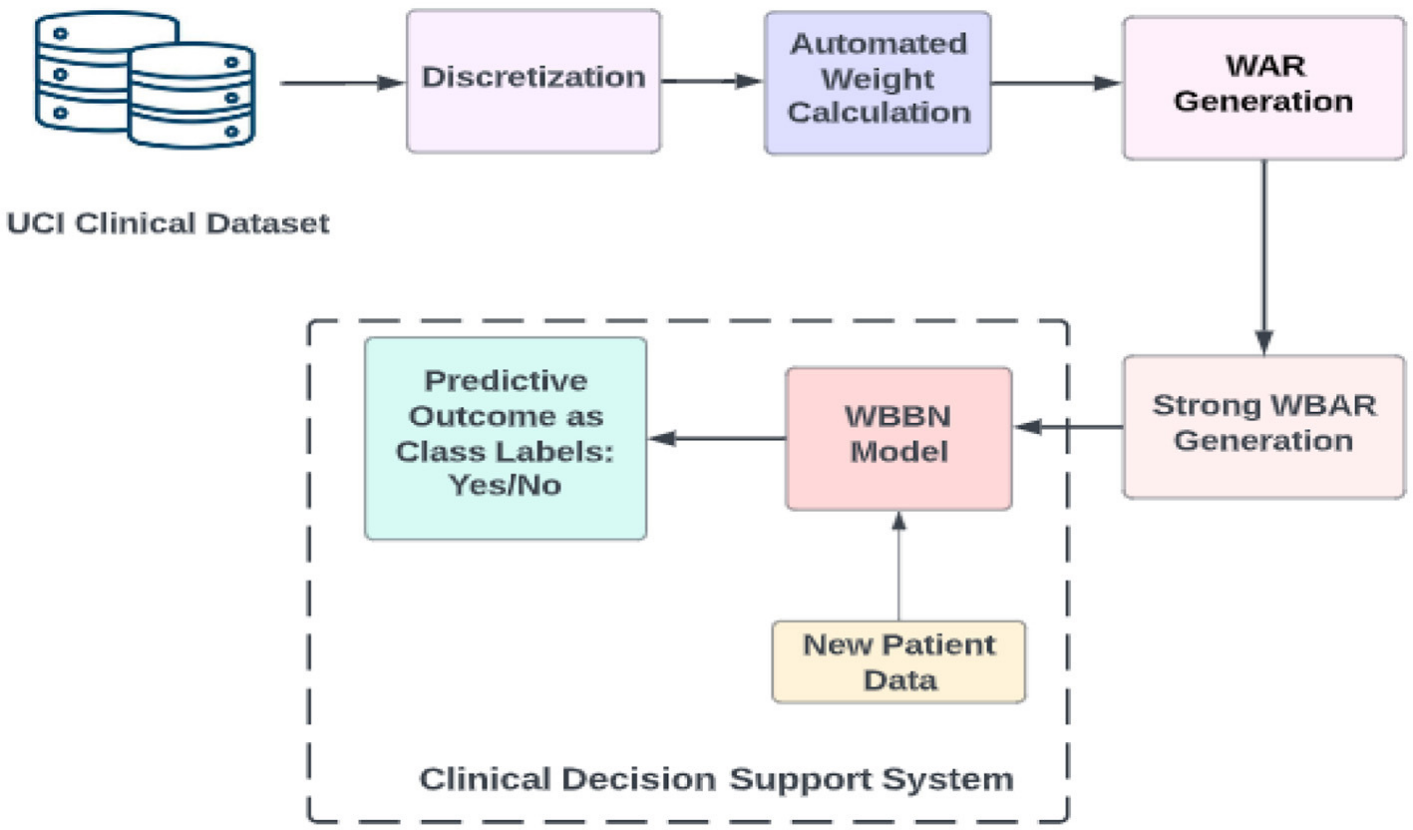
Workflow diagram of the proposed model WBBN.

The procedural approach of the WBBN model proposed in this research is systematically elucidated through a step-by-step representation depicted in the workflow diagram, as shown in Figure 1. This diagram outlines the sequential processes employed in investigating the proposed study.

Following extracting the clinical dataset using the UCI archive and obtaining its discretized form, the subsequent step involves calculating the weights of the {attribute, value} pair using an automated method. Weights can be calculated using domain knowledge-based weight calculation, automated weight calculation, and semi-automated weight calculation (Alwidian et al., 2018). This study calculates weights using the rank-based weight assignment method (Kharya and Soni, 2016). The choice of the rank-based weight assignment method for computing weights of {attribute, value} pairs in the WBBN model can contribute to its robustness and interpretability such as ranking importance, simplicity, and transparency, especially in clinical decision support systems. This ranking clearly indicates which features are most influential in making predictions, enhancing the interpretability of the model. This attribute ranking facilitates clinicians' understanding of the model's decision-making process in clinical decision support systems, where transparency and understandability are crucial.

Different ranking methods may yield varying results in terms of attribute importance. The choice of ranking algorithm or criteria can impact the weights assigned to attribute-value pairs and, consequently, the performance of the model. Careful consideration and validation of the chosen ranking method are necessary to ensure its suitability for the specific clinical domain and dataset.

The whole procedure for building the WBBN model is explained further using definitions, formulas, and pseudocode. The end product is a clinical decision support system used as a predictive model termed the WBBN model.

### 3.1 Weighted bayesian belief network model

The Weighted Bayesian Belief Network classifier comprises a collection of n distinct records, constituting a training dataset T = {r_1_, r_2_, …, r_n_}. Each record constitutes a set of m attributes {a_1_, a_2_, a_3_, ..., a_m_}, with each attribute possessing a unique value v_i_ from its domain, forming a record r_i_ = {v_1_, v_2_, v_3_, ..., v_m_}. In a weighted context, each {attribute, value} pair is assigned a weight, creating a triplet {a_i_, v_i_, w_i_}, where attribute ai with value vi is assigned a weight wi, and 0 < w_i_ ≤ 1, considering value vi as a nominal value. The weight calculated for each attribute implies the significance of the symptoms of the disease. The following section shows how the calculated weights are incorporated into a dataset and how associations between weighted attributes are generated. The application of Bayesian theory to generate robust rules for constructing the proposed model is explained by employing the designed pseudocode.

### 3.2 Definition, formula, and pseudocode

Here, Definitions 1 and 2 explain how to incorporate the weighted concept in the dataset.

#### 3.2.1 Attribute weight

Within a weighted framework, the allocation of weights to attributes is contingent on their predictive capabilities. This study employs a novel method for automatic weight assignment to compute these weights.

#### 3.2.2 Attribute set weight

The weight assigned to the attribute set X is represented as weight (X) and is calculated by determining the average weight of all the constituent attributes through the formula “(1)”.


(1) $$\begin{array}{l} Weight\left(X\right)=\frac{\sum_{i=1}^{\left|x\right|}Weight\left(a_{i}\right)}{No.\;of\;attributes\;in\;X} \end{array}$$

#### 3.2.3 Weight of record

If the dataset comprises n attributes, the record weight is represented as weight (rk), which is calculated by considering the average weight of attributes in the row using equation “(2)”.


(2) $$Weight\;(r_{k})=\frac{\sum_{i=1}^{\left|r_{k}\right|}Weight(a_{i})}{No.\;of\;attributes\;in\;a\;tuple}$$

#### 3.2.4 Weighted support_two attributes

The weighted support between two attributes is calculated using “(3)”.


(3) $$\begin{array}{l} WS\left(A1\to A2\right)=\;\frac{\sum_{i=1}^{A1}\sum_{j=1}^{A2}W\left(r_{ij}\right)}{\sum_{k=1}^{n}W\left(r_{k}\right)} \end{array}$$

#### 3.2.5 Weightedsupport_multi attributes

Within the domain of ARM, the multi-attribute association rules, denoted as A1, A2→A3, represent a specific scenario in association rule mining. In this context, the rule's antecedent plays a determining role in its consequent, where A1, A2, and A3 constitute the set of {attribute, value} pairs. The fraction of weights attributed to records containing the attributes above and values about the total weight of all records is quantified by the calculation outlined in “(4).”


(4) $$\begin{array}{l} WS\left(A1,A2\to A3\right)=\;\frac{\sum_{i=1}^{A1}\sum_{j=1}^{A2}\sum_{k=1}^{A3}W\left(r_{ijk}\right)}{\sum_{k=1}^{n}W\left(r_{k}\right)} \end{array}$$

#### 3.2.6 Weighted support_class

Consider an association rule denoted as X→Y, where weighted support (WS) signifies the proportion of weights assigned to all records containing the specified attributes and value sets relative to the total weight of all records. In this context, X represents a non-empty set of attributes, such as {A1, A2, ..., An}, and Y denotes the class label. The calculation for weighted support can be performed as outlined in “(5).”


(5) $$\begin{array}{l} WS\left(X\to ClassLabel\right)=\frac{\sum_{i=1}^{X}W\left(r_{i}\right)}{\sum_{k=1}^{n}W\left(r_{k}\right)} \end{array}$$

#### 3.2.7 Two attribute's weighted confidence

Suppose the rule A1→A2, with two attributes as A1 and A2, is articulated as the fractional value derived from the weighted support of the two attributes (A1→A2) relative to the weighted support of A1. This relationship is represented as “(6).”


(6) $$\begin{array}{l} WC\left(A1\to A2\right)=\frac{WS\left(A1\to A2\right)}{WS\left(A1\right)} \end{array}$$

#### 3.2.8 Weighted confidence for multi attributes

The rule, such as A1, A2→A3, where A1, A2, and A3 represent multi-attributes, is denoted as the fractional value derived from the weighted support of A1, A2→A3 about the weighted support of A1, A2. This expression is illustrated as “(7).”


(7) $$\begin{array}{l} WC\left(A1,A2\to A3\right)=\frac{WS\left(A1,A2\to A3\right)}{WS\left(A1,A2\right)} \end{array}$$

#### 3.2.9 Weighted confidence for class

It is defined as the fractional value obtained from the weighted support of (X→ClassLabel), where X represents the set of attributes and the weighted support of X. This representation is shown as “(8).”


(8) $$WC\left(X\to ClassLabel\right)=\;\frac{WS(\left(X\to ClassLabel\right)}{WS(X)}$$

Applying the previously stated definitions and formulas, Weighted_class Association rules will be generated. Subsequently, the subsequent definitions and formulas are formulated to compute robust rules for constructing a predictive model rooted in Weighted Bayesian Theory. In this context, WBC and WBL are delineated following the joint probability distributions of the weighted class association rules.

The joint probability distribution for every Weighted_class Association rule is calculated using the BBN standard formula as shown in “(9)”.


(9) $$\begin{array}{l} P\left(A_{1},A_{2},\ldots \ldots .,A_{n}\right)=\overset{N}{\underset{i=1}{\prod }}P\left(A_{i}|Parents\left(A_{i}\right)\right) \end{array}$$

#### 3.2.10 Weighted Bayesian Confidence

The characterization of Weighted Bayesian confidence for the rule A→B, where A represents a set of predictors and B is the class label, is illustrated by the expression P(B|A). This is presented in the following context, as indicated in “(10).”


(10) $$\begin{array}{l} WBC\left(A\to B\right)=P\left(B/A\right)\\ =\frac{WS\left(A,B\right)}{WS\left(A\right)} \end{array}$$

Here, WS(A, B) is the value of joint probability distribution calculated from equation (10). Here, the Weighted Bayesian Confidence value shows the strength of the rules.

#### 3.2.11 Weighted Bayesian Lift

A given rule A→B is characterized as WBC/P(B) and is calculated for the association rule A→B using the Bayesian network, as outlined in “(11)” and “(12).”


(11) $$\begin{array}{l} WBL=\;\frac{WBC}{P\left(B\right)} \end{array}$$

(12) $$\begin{array}{l} \frac{P\left(B/A\right)}{P\left(B\right)}=\;\frac{WS\left(A,B\right)}{WS\left(A\right)WS\left(B\right)} \end{array}$$

The WBL value ranges between 0 (zero) and ∞ (infinity).

If WBL equals to 1, A and B are considered independent.

When WBL is greater than 1, it indicates that the descendant B is positively dependent on the antecedent A, implying a positive correlation between A and B.

If WBL is less than 1, it suggests that the descendant B is negatively dependent on the antecedent A, signifying a negative correlation between A and B (Harpaz et al., 2010; Soni and Vyas, 2013).

Here, Weighted Bayesian Lift values show the correlation between the antecedent and descendent of the rules. The WBBN model is built using two interesting measures, WBC and WBL (Butt et al., 2021; Chang et al., 2022). Bayesian networks inherently deal with uncertainty by modeling probabilistic dependencies between variables. WBC and WBL extend this concept by quantifying the confidence and lift of rules derived from the Bayesian network. This helps account for the uncertainty associated with individual rules and their predictive power. WBC and WBL introduce a weighting mechanism that considers the strength of evidence supporting each rule. This weighting ensures that more reliable and informative rules are given higher importance in the model construction process. By prioritizing rules with higher confidence and lift values, the resulting model becomes more robust and capable of making accurate predictions. By incorporating WBC and WBL metrics into the model construction process, the resulting WBBN model becomes more adept at capturing complex relationships and patterns in the data. Rules with higher confidence and lift values are more likely to accurately represent meaningful associations between variables, thereby improving the predictive power of the model. This leads to more reliable predictions and better performance in real-world applications.

The pseudocode is presented in algorithm to show the clean steps required to generate WBAR to build the predictive model. Weighted Bayesian_**class** Association Rules are extracted using the above formulas and **WBAR Pseudocode** to build the WBBN predictive model using Apriori Algorithm. Here, the procedure Partial_WeightedRule_Generator() is called from WBAR pseudocode to find weighted associations between Two_attributes, Multi_attributes, and finally with Class_labels to generate WARs. After that, the joint probability distribution for all the WARs using eq 9 is calculated. Using the calculated value of each rule, WBC and WBL are computed using “(10)” and eq “(12)”, respectively. WBC shows the reliability or strength of Weighted Bayesian rules, and WBL shows the correlation (positive, negative, and independent) of Weighted Bayesian rules. At last, the model is built using the WBARs with the highest WBC and WBL values.

**Algorithm: WBAR PSEUDOCODE**

**Procedure partial_weightedrule_generator(n,d,a[optional])**

[procedure to extract n attribute partial rules with high weighted confidence over a given dataset Dand A is the Highly associated attribute sets of cardinalities n-1]

1. Most frequent n-attribute sets are extracted as FREQ_ITEMS using the given in_weighted_support_threshold value.
2. For every member L ∈ FREQ_ITEM repeat 2.1 & 2.2
3. Generate all non-empty subsets of L as S

a. For every member X ∈ S Generate the weighted association rules X→L-X and add it RULE_SET
b. For every rule ∈ to RULE_SET, Calculate Weighted_confidence.

4. Partial rules are extracted using the given min_weighted_confidence_threshold and add it to PARTIAL_RULE_SET.
5. For every rule ∈ PARTIAL_RULE_SET of the form p→q, append the consequent attribute q to the antecedent attribute set p to form, attribute set PQ, and add it to the set n- highly_associated_attributes.
6. Return n-highly_associated_attributes.

**Pseudocode: WBAR**

[This algorithm extracts strong Weighted Bayesian_class association rules over a Clinical dataset D with n attributes]

Given Input Data: Database D with n attributes and Binary_ClassLabel.

Outcome Generated: Weighted Bayesian_class Association Rules.

1. Apply discretization on the attributes of D.
2. Apply the automated weight assignment method to assign weights to attributes.
3. Generate weighted 2- highly_associated_attributeset.

a. X[2] = Partial_Weightedrule_generator (2,D)

4. Repeat step 4.1 for k=3,4,.....,n
a. X[k] = Partial_WeightedRule_generator(k,D,X[k-1]) to Generate weighted k - Highly_Associated_AttributeSet

5. Calculate the associations of n-highly_associated_attributesset with classlabel

a. WR= Partial _WeightedRule_generator (n+1,D,X[n])

6. For every w ∈ WR, repeat steps 6.1 and 6.2

a. Calculate the joint probability distribution of w.
b. Calculate the Weighted Bayesian confidence (WBC) and Weighted Bayesian Lift (WBL) for w.

7. To build the model, generate the strong WBAR rules with the highest WBC and WBL.

## 4 Experimental results

A benchmark medical dataset related to PIDD is utilized to assess the effectiveness of the WBBN model by applying weighted Bayesian_class association rules. The construction of the model involves utilizing Java version 1.8 for the front end and MySql 8 as the backend tool. Different proportions of the dataset are employed to train and test to learn the innovative predictive model, employing various thresholds for Min_Weighted support and Min_Weighted confidence. The outcomes of four distinct experimental scenarios are tabulated using Tables 3–6. In this study, the WBBN model undergoes thorough training and testing using a distinct distribution of the PIDD dataset comprising 768 records. The primary evaluative parameter employed in this study is accuracy, which is the correctness of predictions made by a predictive model for diabetes diagnosis or classification (Nayak et al., 2023; Panigrahi et al., 2023; Pati et al., 2023).

**Table 3:** Generation of strong rules based on WBC and WBL when min_WS = 10% and min_WC = 50% with achieved accuracy.

**S.No**	**Minimum threshold on weighted approach**	**Training dataset**	**Testing dataset**	**No.of rules based on WS and WC (WARs)**	**No. of strict rules based on WBC and WBL (WBARs)**	**Accuracy**
1	Support = 10%; Confidence = 50%	100%	100%	24	15	89.53
2		80%	20%	20	12	92
3		70%	30%	15	10	91
4		60%	40%	22	14	90

**Table 4:** Generation of strong rules based on WBC and WBL when min_WS = 40% and min_WC = 80% with achieved accuracy.

**S.No**	**Minimum threshold on weighted approach**	**Training dataset**	**Testing dataset**	**No.of rules based on WS and WC (WARs)**	**No. of strong rules based on WBC and WBL (WBARs)**	**Accuracy**
1	Support = 40%; Confidence = 80%	100%	100%	10	5	89
2		80%	20%	12	7	95.8
3		70%	30%	10	7	85
4		60%	40%	24	10	92

**Table 5:** Generation of strong rules based on WBC and WBL when min_WS = 26% and min_WC = 60% with achieved accuracy.

**S.No**	**Minimum threshold on weighted approach**	**Training dataset**	**Testing dataset**	**No.of rules based on WS and WC (WARs)**	**No. of strong rules based on WBC and WBL (WBARs)**	**Accuracy**
1	Support = 26%; Confidence = 60%	100%	100%	20	10	89.53
2		80%	20%	22	11	93
3		70%	30%	21	10	90
4		60%	40%	10	7	92.55

**Table 6:** Generation of strong rules based on WBC and WBL when min_WS = 36% and min_WC = 70% with achieved accuracy.

**S.No**	**Minimum threshold on weighted approach**	**Training dataset**	**Testing dataset**	**No.of rules based on WS and WC (WARs)**	**No. of strong rules based on WBC and WBL (WBARs)**	**Accuracy**
1	Support = 36% Confidence = 70%	100%	100%	20	10	92.3
2		80%	20%	10	6	94
3		70%	30%	9	4	93
4		60%	40%	9	6	92.5

### 4.1 Minimum threshold setup

Examining the significance of the Minimum Threshold value (Min_Thres) about weighted support and weighted confidence, these factors directly impact the accuracy of the classifier model outcomes. If the Min_Thres is set too low, it may include irrelevant rules in the rule base. Conversely, setting the Min-Thres too high may result in excluding valuable and essential rules that exhibit high confidence (Chang et al., 2022). Moreover, the model is empirically tested by setting different threshold values to acquire the highest accuracy. Initially, the generation of Weighted Association Rules (WARs) involves the consideration of minimum threshold values for Weighted Support (WS) and Weighted Confidence (WC) across two attributes, multi-attributes, and incorporating a class label. This process follows the steps delineated in the provided pseudocode. Subsequently, the construction of the Weighted Bayesian Belief Network (WBBN) model is achieved by utilizing Weighted Bayesian Confidence (WBC) and Weighted Bayesian Lift (WBL) to generate robust rules. Finally, using this strong rule model, WBBN is trained. Test data are applied to the model to check the accuracy of the WBBN model, and its achieved accuracy is also presented in the following tables. The experimental outcomes, detailing the generation of robust rules and their respective accuracies, are tabulated using Tables 3–6.

**Figure 2:**
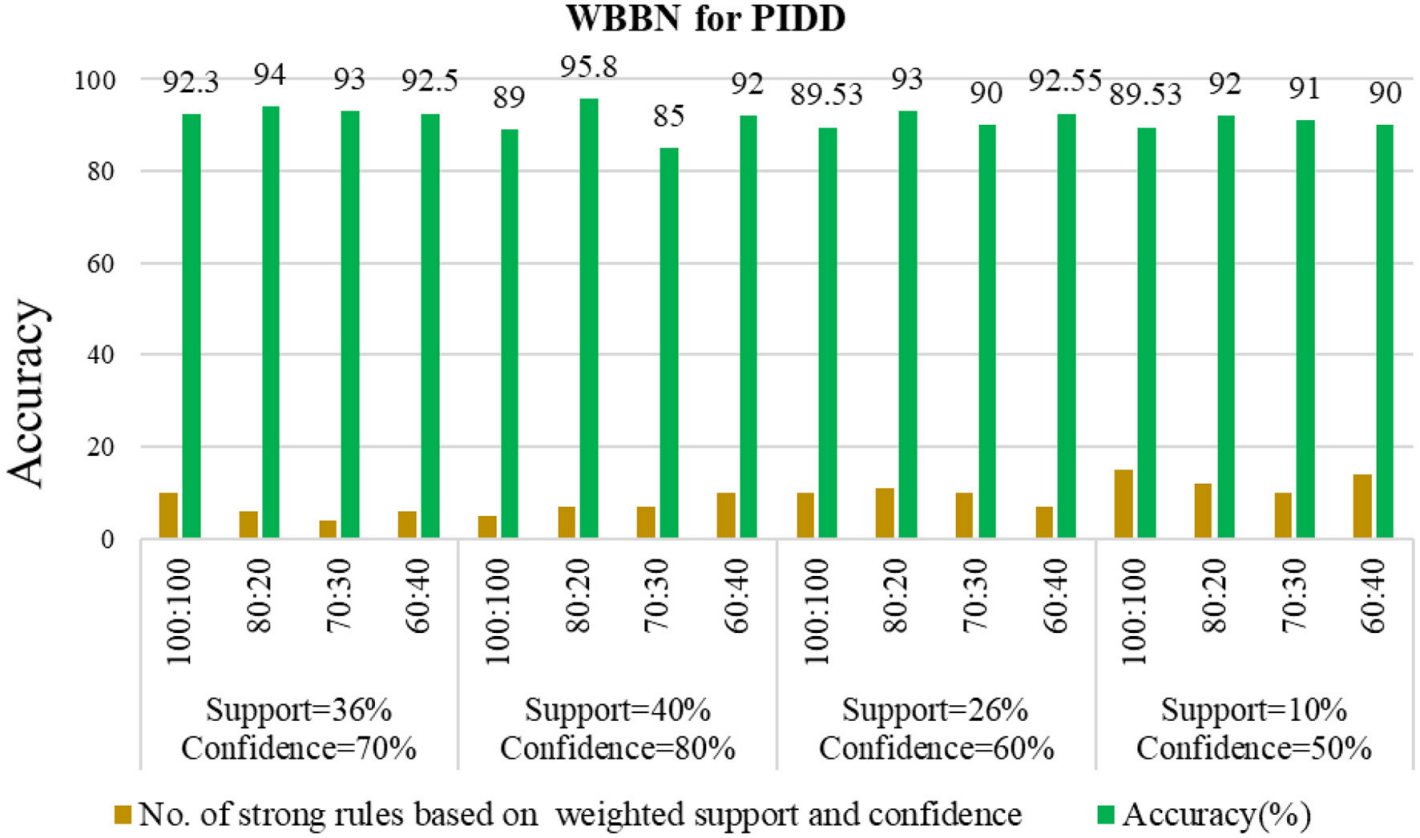
Empirical analysis of WBBN using pidd on different parameters.

The experimental setup shows that seven strict rules are generated to develop the model when WBBN is trained with an 80% training dataset (614 records). Then, to check the accuracy, it is tested using test data of 20% (154 records); the highest accuracy acquired is 95.8% with Minimum WS = 40% and WC = 80%, as shown in Table 4. Moreover, as a model should be built using a minimum number of strong rules, WBBN uses seven rules. The graphical representation of the results is shown in Figure 2, which gives the highest accuracy of 95.8 % for the PIDD dataset.

Now, this WBBN model is ready for class label prediction. When new patient data are fed to the model, it checks with the strong rules and assigns the class label accordingly.

## 5 Comparative study

**Figure 3:**
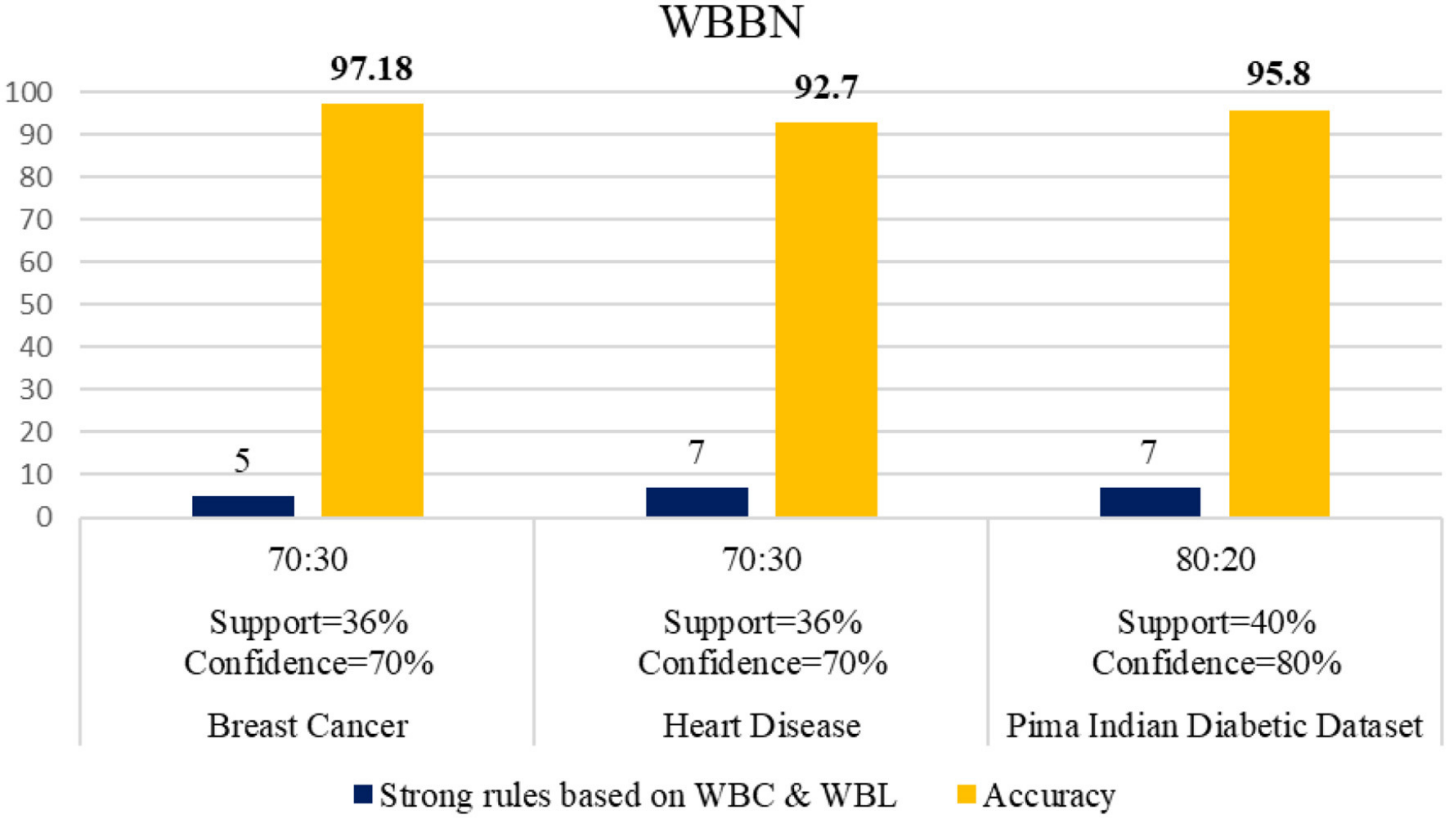
Performance of WBBN on various clinical datasets.

The WBBN model using the clinical dataset is evaluated regarding the number of strong rules and accuracy. Table 7 shows the results of the WBBN model using three clinical datasets: the Pima Indian Diabetic dataset, the Heart Disease dataset, and the Breast Cancer dataset.WBBN model, built using seven strong rules of PIDD, achieves an accuracy of 95.8% when the model is trained using 80% of the dataset, and tested on 20% with provided Min_Threshold. Again, the WBBN model built using seven strict rules of the heart disease dataset acquires an accuracy of 92.7% when the model is trained on 70% of the dataset and tested on 30% of the data with the provided Min_Threshold. Similarly, the WBBN model achieves the highest accuracy of 97.18%, as shown in Table 7. The graphical representation of the results is shown in Figure 3.

**Table 7:** Performance of WBBN on various clinical datasets.

**Datasets**	**Min. weighted threshold**	**Data distribution**		**No. of strong rules based on WBC and WBL**	**Accuracy (%)**
		**Training dataset**	**Test dataset**		
Pima Indian Diabetic Dataset	Support = 40%; Confidence = 80%	80%‘	20%	7	95.8
Heart Disease Dataset	Support = 36%; Confidence = 70%	70%	30%	7	92.7
Breast Cancer	Support = 36%; Confidence = 70%	70%	30%	5	97.18

Now, the exhaustive comparison of the proposed model WBBN is done with existing classifiers developed in recent years using the same diabetic dataset. The comparative study shows that the proposed model WBBN gives outstanding results, as shown in Table 8. In this comparison, the WBBN model achieves the highest accuracy of 95.8%. This could be attributed to its ability to capture complex relationships between features in the dataset. The superior performance of WBBN indicates the potential effectiveness of probabilistic modeling for classification tasks.

**Table 8:** Performance comparison of wbbn with existing classifiers on the pima Indian diabetic dataset.

**Dataset**	**Classifier model**	**Accuracy (%)**
PIDD	WBBN (proposed model)	95.8%
	Naïve Bayes (Chang et al., 2022)	80%
	SVM (Patil et al., 2022)	94.5%
	MLP (Butt et al., 2021)	86.08%
	ANN (Jader and Aminifar, 2022)	91%
	Decision tree (Azad et al., 2022)	82.12%

Here, Naive Bayes achieves an accuracy of 80%, which is notably lower than WBBN. The simplicity of Naive Bayes, relying on the assumption of feature independence, may not fully capture the complex relationships in the dataset, leading to lower accuracy.

Again, SVM performs admirably with an accuracy of 94.5%, slightly below WBBN. SVM's ability to identify complex decision boundaries in high-dimensional spaces might contribute to its competitive performance. MLP and ANN achieve accuracies of 86.08% and 91%, respectively.

While these neural network models demonstrate reasonable performance, they fall short of WBBN, possibly due to suboptimal architecture or training parameters. Decision Tree achieves an accuracy of 82.14%, which is relatively lower than other models. Inherent limitations of Decision Trees in capturing complex relationships and tendency to overfit might contribute to their lower accuracy.

**Figure 4:**
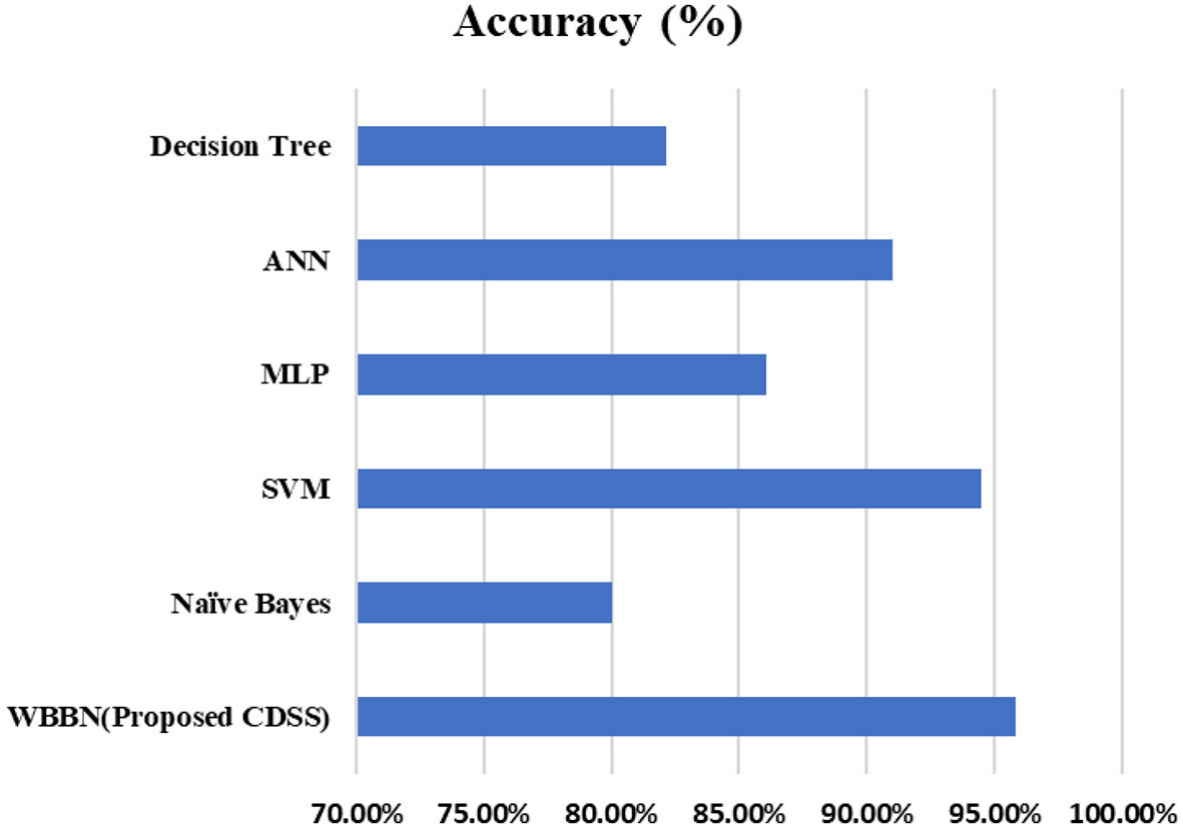
Comparison of WBBN with other classifiers using the pid dataset.

To increase the visualization of a comparison, a graph has been plotted for PIDD clinical datasets on various existing classifiers such as Naïve Bayes, SVM, MLP-NN, K-NN, Random forest, and Decision Tree, which are commonly used in the clinical industry, as shown in Figure 4. In conclusion, while WBBN demonstrates superiority in accuracy over other classifiers on the Pima Indian Diabetic Dataset, further research is needed to explore its interpretability, scalability, and generalization capabilities. Additionally, addressing the limitations and challenges encountered during the evaluation processes, such as dataset bias and class imbalance, would provide valuable insights for enhancing the effectiveness of classifiers in practical applications, particularly in healthcare and related domains.

## 6 Conclusion and future work

The construction of the WBBN model involves incorporating the property of the clinical dataset, specifically the notion that “not all symptoms are equally important for prediction.” This is achieved by assigning varying weights to attributes based on their predictive capabilities. The experimental findings demonstrate that the weighted concept contributes to achieving higher accuracy in the clinical domain compared with other available predictive models. The experiments were conducted using three distinct clinical datasets, namely, the Breast Cancer dataset, Heart Disease dataset, and PIDD from the UCI archive, yielding outstanding results. This achievement represents a significant contribution to the medical sector. In the future, the innovative weighted model could be extended to non-clinical datasets to benefit other sectors. Extending the weighted model to non-clinical datasets requires careful consideration of domain-specific characteristics, data quality, feature engineering, interpretability, generalization, ethical considerations, and scalability. While the weighted model may offer advantages in terms of performance and interpretability, addressing the challenges and limitations inherent in applying the model to diverse datasets beyond the medical domain is essential for its successful adoption and deployment in non-clinical sectors. Additionally, addressing the “Sharp Boundary problem in the medical field” could be achieved by incorporating fuzzy theory and developing a fuzzy weighted model.

## Data availability statement

The original contributions presented in the study are included in the article/supplementary material, further inquiries can be directed to the corresponding authors.

## Author contributions

SK: Writing—original draft, Writing—review & editing. SS: Writing—original draft, Writing—review & editing. APat: Conceptualization, Investigation, Writing—review & editing, Writing—original draft. APan: Conceptualization, Investigation, Writing—review & editing, Writing—original draft. JG: Conceptualization, Data curation, Investigation, Methodology, Visualization, Writing—review & editing, Writing—original draft. HQ: Conceptualization, Data curation, Investigation, Methodology, Software, Writing—original draft, Writing—review & editing. SM: Conceptualization, Investigation, Software, Writing—review & editing, Writing—original draft. DN: Data curation, Investigation, Writing—review & editing, Writing—original draft. TS: Conceptualization, Data curation, Writing—review & editing, Writing—original draft.
